# Hepatitis B Virus Reactivation upon Immunosuppression: Is There a Role for Hepatitis B Core-Related Antigen in Patients with Immune-Escape Mutants? A Case Report

**DOI:** 10.3390/diagnostics11122185

**Published:** 2021-11-24

**Authors:** Gian Paolo Caviglia, Antonella Zorzi, Mario Rizzetto, Massimo Mirandola, Antonella Olivero, Giada Carolo

**Affiliations:** 1 Department of Medical Sciences, University of Turin, 10126 Turin, Italy; mario.rizzetto@unito.it; 2 Virology and Microbiology Unit, Department of Pathology and Diagnostics, Verona University Hospital, 37126 Verona, Italy; antonella.zorzi@aovr.veneto.it; 3 Infectious Diseases Section, Department of Diagnostics and Public Health, University of Verona, 37134 Verona, Italy; massimo.mirandola@univr.it (M.M.); giada.carolo@aovr.veneto.it (G.C.)

**Keywords:** HBcrAg, HBsAg, HBV, HBV DNA

## Abstract

The reactivation of hepatitis B virus (HBVr) in patients undergoing pharmacological immunosuppression is a potentially fatal clinical event that may occur in patients with overt or occult HBV infection. The risk of HBVr is mainly determined by the type of immunosuppressive therapy and the HBV serologic profile, with a higher risk in patients positive for the hepatitis B surface antigen (HBsAg), and a lower risk in HBsAg-negative/antibodies to core antigen-positive subjects. Notably, a considerable proportion of patients experiencing HBVr showed a high degree of variability of the HBV S gene, possibly leading to immune escape mutants. These mutations, usually in the “a-determinant” of the HBsAg, can cause diagnostic problems and consequently hamper the appropriate management strategy of patients at risk of HBVr. Here, we describe a case of HBVr in a patient with a diagnosis of chronic myeloid leukemia and a previous history of kidney transplant, providing evidence of the potential usefulness of hepatitis B core-related antigen measurement in patients with HBV immune-escape mutants at risk of viral reactivation.

## 1. Introduction

Hepatitis B virus reactivation (HBVr) encompasses a wide spectrum of clinical conditions ranging from an asymptomatic increase in circulating HBV DNA to hepatitis flares and even acute liver failure [1]. HBVr can occur both in hepatitis B surface antigen (HBsAg)-carriers or in subjects with a resolved HBV infection; the risk is primarily determined by the duration and potency of pharmacological immunosuppression [2].

HBV covalently closed-circular (ccc)DNA is the replicative intermediate of HBV responsible for the persistence of the virus within the hepatocytes [3]. HBV cccDNA acts as a template for all viral transcripts, including sub-genomic RNAs, pre-core RNA and pre-genomic RNA, that encode for the structural and soluble proteins of HBV [4]. Following the resolution of HBV infection, the replicative activity of HBV cccDNA is inhibited by the surveillance pressure of a competent innate and adaptative immune response; however, it can silently persist for decades in the liver of HBsAg-negative subjects with serum markers of past HBV exposure, a condition referred to as occult HBV infection [5,6]. 

Patients with occult HBV infection and hematological malignancies receiving cytotoxic or immunosuppressive therapies are at risk of HBVr [7]. Furthermore, HBVr is usually associated with the emergence of immune-escape mutations within the HBV S gene (i.e., the HBV gene that encodes for the HBsAg) that confers the virus a significant survival advantage, while hampering circulating HBsAg detection using commercial serological assays [8]. A recent study pointed out a high prevalence of HBV S gene mutations in patients with malignant blood diseases, reporting up to 15 different mutations in a single patients’ isolate [9]. 

Recently, Seto and colleagues investigated risk factors associated to HBVr in oncohematological patients with resolved HBV infection undergoing rituximab-based chemotherapy or allogeneic hematopoietic stem cell transplantation [10]. Interestingly, the authors observed that higher baseline hepatitis B core-related antigen (HBcrAg) levels were independently associated to HBVr, suggesting a potential clinical value of the biomarker for the identification of patients that might benefit from prophylactic nucleos(t)ide analogue (Nuc) treatment [10].

Here, we describe a case of reactivation of Hepatitis B virus with multiple gene S mutations, providing evidence for the possible clinical value of the measurement of the HBcrAg in immunosuppressed patients at risk of viral reactivation.

## 2. Case Report

In July 2019, a 74-year-old male patient was admitted to the Infectious Disease Section of the Verona University Hospital for investigation; he had HBV in his blood with a titer of 26,100,000 IU/mL (cobas^®^ HBV, Roche Molecular Diagnostics, Branchburg, NJ, USA) but exhibited normal liver function. Although he displayed the hepatitis B e antigen (HBeAg), he was negative for the HBsAg, but positive for the homologous anti-HBs (ADVIA Centaur HBV assays, Siemens Healthcare GmbH, Erlangen, Germany).

In August 2013, the patient received a kidney transplant for nephroangiosclerosis. At this time, the serologic screening for HBV had shown that he was HBsAg-negative, anti-HBs-positive (12 mIU/mL), HBeAg-negative and positive for antibodies to the HBeAg and to the hepatitis B core antigen (anti-HBc). No antibody markers of a hepatitis C and hepatitis D virus infection were detected; serum HBV DNA had not been determined. The indices of hepatic cytolysis were normal. The kidney donor was negative for HBV. Post-transplant, the patient received immunosuppressive induction with basiliximab, tacrolimus, mycophenolate and steroids, and was then included in the follow-up program as per protocol; the HBsAg remained negative throughout, accompanied by normal liver biochemistry.

**Figure 1:**
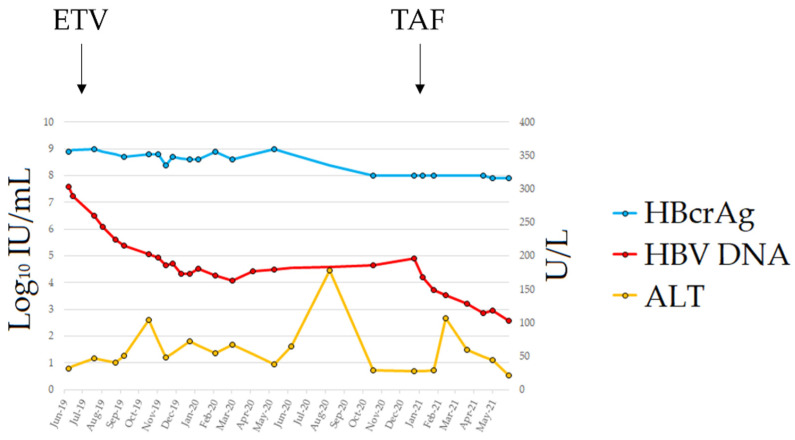
Clinical course from HBVr diagnosis to last follow-up. Abbreviations: alanine aminotransferase (ALT); entecavir (ETV); hepatitis B core-related antigen (HBcrAg); hepatitis B surface antigen (HBsAg); tenofovir alafenamide (TAF).

In May 2019, the patient developed chronic myeloid leukemia (CML). Before treatment with imatinib mesylate, the patient repeated the HBV serology and the HBeAg was again detected in his blood in the absence of circulating HBsAg; further testing using a real-time PCR showed that he had HBV DNA in serum at a titer of 26,100,000 IU/mL. The patient was still anti-HBs-positive (15 mIU/mL) with normal liver enzymes. A diagnosis of HBVr was made and, in July 2019, the patient started entecavir (ETV) treatment at 0.5 mg/day, which was subsequently reduced in April 2020 to 0.5 mg/48 h because of renal function deterioration. The patient underwent a close follow-up (every 2 weeks) for the first 6 months of treatment and, after, with monthly scheduling. The therapy led to a consistent decrease in viremia, reaching a plateau of 3 Log_10_ HBV DNA reduction after 6 months of treatment (Figure 1); however, viremia did not decrease further in the following months. No classic ETV-resistant mutations were observed in the HBV reverse transcriptase (rt) gene. However, we detected the mutation rtL269I, previously reported to confer a reduced susceptibility to ETV [11]. On January 2021, the patient switched to tenofovir alafenamide (TAF) treatment; HBV DNA rapidly declined and reached a value of 378 IU/mL at the last FU. The single ALT elevation (107 U/L) in the 2nd month of TAF treatment was documented in the course of an infection of the residual left limb requiring surgical toileting. Unfortunately, the patient died from coronary heart disease in September 2021.

The serum samples collected from July 2019 were tested for the HBsAg with the highly sensitive Lumipulse^®^ G HBsAg-Quant assay (Fujirebio Inc., Tokyo, Japan) as well as for the HBcrAg (Lumipulse^®^ G HBcrAg, Fujirebio Inc., Tokyo, Japan) [12]. The nucleotide sequences of the HBV S gene and the rt domain were determined using the Sanger sequencing method at BMR Genomics service (BMR Genomics, Padua, Italy) and analyzed using ClustalW2 software (https://www.ebi.ac.uk/Tools/msa/clustalw2/; accessed on 23 December 2019 and on 6 December 2020) [13]. The HBV genotype was determined based on a reverse hybridization line probe assay (INNO-LiPA HBV Genotyping, Fujirebio Europe, Gent, Belgium) [14].

**Figure 2:**
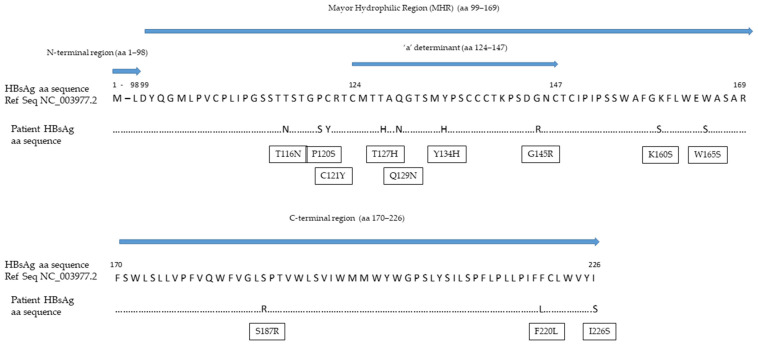
Mutations within the HBV S gene detected in our patient. Abbreviations: amino acid (aa); hepatitis B surface antigen (HBsAg).

Despite its high analytical sensitivity for HBsAg mutants [15], the HBsAg assay failed to detect a circulating HBs antigen. The HBV S gene sequence analysis identified four mutations within the a-determinant (aa 124–147) (P/T127H, Q129N, F/Y134H and G145R) and five additional mutations within the mayor hydrophilic region (MHR, aa 99–169) (T116N, P120S, C121Y, K/R160S and W165S) (Figure 2). The values of the HBcrAg from baseline to the end of the follow-up were persistently above the upper limit threshold of the assay (>7.0 Log_10_ U/mL). Viral genotyping showed that the patient was infected with HBV genotype D.

## 3. Discussion

HBsAg-negative/anti-HBc-positive patients undergoing immunosuppressive therapy following a solid organ transplant or tyrosine kinase inhibitors are at moderate risk of HBVr (from 1% to 10%) [16]; in these patients, pre-emptive therapy, rather than HBV prophylaxis, is recommended [17]. In case of HBsAg seroreversion, treatment with Nucs should be started as early as possible, independently from ALT levels [17]. However, in our patient, as well as in several other cases reported in the literature [18,19,20,21,22,23], HBVr occurred in default of the detectable HBsAg in serum. In this regard, the search for escape mutants should be recommended in patients with CML and markers of previous HBV exposure when undergoing pharmacological immunosuppression.

As far as the viral sequencing is concerned, we observed a high degree of HBV S gene variability accompanied by several mutations responsible for the changes in the antigenic pattern of the HBsAg. These mutations can prevent both neutralizing and diagnostic anti-HBs from interacting with the HBsAg; as a result, it is not uncommon to find anti-HBs antibodies in patients experiencing HBsAg-negative HBVr [24]. 

In our patient, four mutations were observed within the a-determinant. These included the most common HBV immune escape mutation at position 145, present as a glycine to arginine change, and another three mutations at positions 127, 129 and 134, which are frequently observed in patients with HBVr and known to be responsible for changes in the loop structure of the HBsAg [25]. Among the mutations observed outside the a-determinant but within the MHR, P120S can alter the antigenicity of the HBsAg by interfering with the loop structure of the a-determinant, while T116M is responsible for the introduction of additional N-linked glycosylation sites that can mask B-cells epitopes and, thus, increase HBV evasion from the humoral immune response [18,25]. Remarkably, the rate of mutations observed in our case was so high that the detection of circulating HBsAg by the most sensitive commercial assay was inhibited [15]. Nonetheless, we recommend the use of highly sensitive HBsAg quantification methods with high reactivity against HBV S gene mutants. A previous study showed an excellent analytical sensitivity for the Lumipulse^®^ G HBsAg-Quant assay; among 1000 seronegative subjects, the assay detected 29 serum samples positive for the HBsAg, with concentrations between 0.005 and 0.05 IU/mL [26]. Furthermore, the Lumipulse^®^ G HBsAg-Quant assay proved higher reactivity against the HBsAg mutants as compared to other commercial assays; the analytical method involves the HBsAg denaturation in linear form, allowing for the full exposure of the inner and outer portion of the a-determinant, thus increasing the number of epitopes that can be recognized by capture antibodies [15]. In our case, we suspected HBVr from the seroreversion of the HBeAg; this prompted the molecular testing, which revealed a highly replicative virus in the blood with an HBV DNA concentration of 26,100,000 IU/mL. HBVr was not recognized before, because national and international guidelines [17,27] do not recommend the assessment of HBV DNA in HBsAg-negative/anti-HBc-positive patients at moderate risk of HBVr. 

To avoid overlooking HBV replication, a simple and cheaper alternative is the measurement of the HBcrAg. This is a composite biomarker that simultaneously detects the hepatitis B core antigen (HBcAg), the HBeAg and the core-related protein, p22cr [28]. These three core-related proteins share an identical 149 amino acid sequence and are expressed by the pre-core/core HBV gene [29]. The HBcAg is the major structural protein of the HBV capsid. The HBeAg is a soluble non-structural pre-core protein, while p22cr is an aberrantly processed pre-core protein that lacks the C-terminal arginine-rich domain responsible for HBV genome packaging [30]. The HBcrAg is measured in serum samples through a fully automated procedure; currently, it is considered the most reliable surrogate for intrahepatic HBV cccDNA quantity and transcriptional activity [12,31].

In conclusion, considering the economic burden of serial HBV DNA assessment and given the high correlation between HBV DNA and the HBcrAg [32], the combined use of the highly sensitive HBsAg and HBcrAg may represent a cost-effective monitoring option for HBsAg-negative/anti-HBc-positive patients with low-to-moderate risk of HBVr.

## Data Availability

The data presented in this study are available upon request from the corresponding author.
